# Buprenorphine implementation at syringe service programs following waiver of the Ryan Haight Act in the United States

**DOI:** 10.1016/j.drugalcdep.2022.109504

**Published:** 2022-05-20

**Authors:** Barrot H. Lambdin, Ricky N. Bluthenthal, Hansel E. Tookes, Lynn Wenger, Terry Morris, Paul LaKosky, Alex H. Kral

**Affiliations:** a RTI International, Berkeley, CA, United States; b University of California San Francisco, San Francisco, CA, United States; c University of Washington, Seattle, WA, United States; d University of Southern California, Los Angeles, CA, United States; e University of Miami, Miami, FL, United States; f North American Syringe Exchange Network, Tacoma, WA, United States

**Keywords:** Buprenorphine, Syringe service programs, Telehealth, Policy, Ryan Haight Act, People who use drugs, Opioids

## Abstract

**Introduction:**

Among people with an opioid use disorder in the United States, only 10% receive buprenorphine treatment. The Ryan Haight Act is a federal law that has regulated buprenorphine delivery, requiring an in-person examination between a patient and provider before initiating treatment. At the beginning of the COVID-19 pandemic, federal agencies waived in-person examination requirements for buprenorphine treatment initiation. We examined whether Ryan Haight Act waiver improved implementation of telehealth buprenorphine within syringe service programs (SSPs) – organizations that serve people with historically low access to treatment.

**Methods:**

We surveyed all known SSPs operating in the US in 2021 (N = 421) of which 77% responded (n = 325). We calculated the prevalence and accompanying 95% confidence intervals (CI) for implementation of telehealth buprenorphine inductions at SSPs in 2020. Multivariable logistic regression was used to assess differences in implementing telehealth buprenorphine inductions by organizational characteristics.

**Results:**

In 2020, the prevalence of implementing buprenorphine inductions via telehealth was 24% (95% CI:19–30%). Non-governmental SSPs had a higher odds of telehealth buprenorphine inductions (adjusted odds ratio (aOR) = 2.92; 95% CI:1.22–7.00; p = 0.016), compared to governmental SSPs. Furthermore, the larger the organization’s annual budget, the higher the odds of telehealth buprenorphine implementation (aOR = 2.00 per quartile (95% CI:1.33–2.99; p = 0.001). SSPs located in states with higher opioid overdose mortality rates did not have significantly higher likelihood of telehealth buprenorphine implementation.

**Conclusion:**

A substantial number of SSPs implemented telehealth buprenorphine after waiver of the Ryan Haight Act. Permanent adoption of this waiver will be critical and providing financial resources to SSPs is vital to support implementation of new innovations.

## 1. Introduction

An estimated 6.7–7.6 million adults have an opioid use disorder (OUD) in the United States (US), with less than 20% of people receiving medications for their OUD (MOUD) (Keyes et al., 2022). Buprenorphine, a MOUD approved by the US Food and Drug Administration in 2002, has a strong evidence-base and has been shown to reduce opioid overdose mortality (US Department of Health and Human Services, 2020; Sordo et al., 2017). Of the approved MOUDs, buprenorphine is considered to have the greatest opportunity for expanded access in the US, because it does not require patients to visit specialty clinics on a daily basis (US Department of Health and Human Services, 2020; Haffajee et al., 2018). However, major barriers exist in ensuring access, as only 10% of people with an OUD are receiving buprenorphine (Anderson et al., 2021; Olfson et al., 2020; SAMHSA, 2020). A recent US study combining data from the National Ambulatory Medical Care Survey and the National Hospital Ambulatory Medical Care Survey from 2004 to 2015 showed that White patients had considerably higher levels of buprenorphine prescription than patients of other races/ethnicities, with Black patients having significantly lower odds of receiving buprenorphine prescription at their visits (Lagisetty et al., 2019). Self-pay and private insurance were the most common payment methods (Lagisetty et al., 2019).

Since 2008, the Ryan Haight Act has required a patient to have an in-person examination with a trained provider before buprenorphine can be initially prescribed to someone with an OUD (Title, 2020; Wenger et al., 2021); in effect, this requirement has limited buprenorphine access throughout the US (Andrilla et al., 2017). In concert with other federal agencies, the US Drug Enforcement Agency waived the in-person examination requirement to prescribe buprenorphine in March 2020 at the beginning of the COVID-19 pandemic (Title, 2020; US Department, 2020). With this waiver, medical providers could prescribe and induct patients onto buprenorphine through virtual or telephone platforms without a urine drug screen, signed treatment agreement or paper prescription. This waiver simplified the process for initiating buprenorphine treatment for OUD (Title, 2020; US Department, 2020).

Syringe service programs (SSPs) have been a linchpin for public health efforts to prevent and treat HIV, HCV, and overdose fatalities among people who use drugs (Lambdin et al., 2020a). At their core, SSPs provide access to and disposal of sterile syringes and injection equipment for people who inject drugs (CDC. Syringe, 2020). With over 400 operating throughout the United States, SSPs are also ideal venues to offer buprenorphine treatment – potentially serving a key role of connecting interested participants who have an OUD to trained providers. Yet, limited geographic coverage of trained providers has been a major impediment for buprenorphine implementation historically (US Department of Health and Human Services, 2020). Waiver of the in-person examination requirements for buprenorphine treatment facilitated new possibilities for streamlined buprenorphine treatment delivery at SSPs. We assessed whether waiver of the Ryan Haight Act led to implementation of telehealth buprenorphine treatment at SSPs as well as which organizational characteristics were associated with telehealth buprenorphine implementation throughout the United States.

## 2. Materials and methods

### 2.1. Study setting and design

We conducted a cross-sectional study of all SSPs known to be operating in the United States in 2021. As newly opening SSPs would be less likely to have the capacity to adapt with this US policy shift, we excluded SSPs that opened in 2020 or 2021.

Our study procedures were reviewed by internal review board within the Office of Research Protection at RTI International’s internal review board (STUDY00021210). While this research was determined to be exempt under 45 CFR 46.104 (d)(2)(ii), the study did include a consent process which disclosed the activities of the research, the procedures to be performed, that participation was voluntary, the investigator name and contact information and the provisions to maintain the privacy of subjects. Because the study was determined to be exempt, the consent process did not require a written or electronic signature or a waiver of written documentation of consent. However, participants had to indicate their consent to participate by selecting a button on the webpage before advancing into the online survey.

### 2.2. Data collection procedures

We recruited SSPs operating throughout the United States to complete an online survey using the Voxco© platform (Voxco, Montreal, Quebec, Canada) from February to June 2021. To do so, we followed previously described procedures (Lambdin et al., 2020a). First, the North American Syringe Exchange Network emailed SSP contacts from their database of SSPs operating in the United States, developed and maintained over the past 30 years. SSPs were emailed up to three times asking organizational directors to participate in an online survey. Next, we conducted additional follow-up with individual programs via email and/or phone calls if a program had not responded. SSPs were offered a $75 honorarium if they completed the survey. The survey took a median of 18 min to complete (interquartile range: 11, 34 min) and included questions with regards to organizational characteristics, syringe distribution, naloxone distribution, fentanyl test strip distribution and buprenorphine treatment implementation.

We also utilized publicly available data regarding opioid overdose deaths from the National Center for Health Statistics (Centers for Disease Control, 2021). Using the International Classification of Diseases, Tenth Revision (ICD-10), opioid overdose mortality rates (OOMRs) were identified using ICD-10 codes X40–X44; X60–X64; X85; or Y10–Y14, where the multiple cause of death codes also included T40.0, T40.1, T40.2, T40.3, T40.4 or T40.6. We aggregated OOMRs for each state. We utilized overall OOMRs as well as OOMRs for American Indian/Native Alaskan, non-Hispanic Black, Hispanic and non-Hispanic White populations. For overall OOMRs, we used data from 2019. For American Indian or Alaskan Native, non-Hispanic Black, Hispanic, and non-Hispanic white OOMRs, we used data from the five-year period of 2015–2019 to ensure sufficient numbers for stable rate calculations.

### 2.3. Measures

Our primary outcome variable was a dichotomized variable of whether the SSP implemented telehealth – audio-only or audio-visual – buprenorphine inductions during 2020. On January 31, 2020, a public health emergency was declared by the US Department of Health and Human Services (DHHS) due to the COVID-19 pandemic, and on March 16, 2020, DHHS with concurrence from the Drug Enforcement Administration declared that the telehealth allowance applied to all schedule II-IV controlled substances, including buprenorphine, in all areas of the United States (Title, 2020; US Department, 2020). Given that telehealth buprenorphine inductions were not legally possible prior to waiver of the Ryan Haight act, our underlying assumption is that telehealth buprenorphine inductions that were implemented in 2020 did so after the waiver of the Ryan Haight Act took effect.

Our exposures of interest included: type of SSP – a dichotomous variable of whether the SSP was a non-governmental organization or whether the SSP was operated by local, county or state government; census regions – a categorical variable the SSP’s location within different census regions (West, Midwest, South, Northeast) as defined by the United States census bureau; annual budget – the SSP’s previous year’s annual budget which was a continuous variable; state-level opioid overdose death rates from 2019; state-level opioid overdose death rates among American Indian or Alaskan Native from 2015 to 2019; state-level opioid overdose death rates among non-Hispanic Black people from 2015 to 2019; state-level opioid overdose death rates among Hispanic people from 2015 to 2019 and state-level opioid overdose death rates among non-Hispanic White people from 2015 to 2019.

### 2.4. Statistical analysis

Descriptive statistics, including frequencies, median and interquartile range, were calculated to describe the distribution of variables in the study population. We calculated the prevalence and accompanying 95% confidence intervals (CI) for implementation of telehealth buprenorphine inductions at SSPs in 2020. Because SSPs could not offer telehealth buprenorphine inductions prior to the Ryan Haight waiver in March 2020, all telehealth buprenorphine implementations in 2020 were considered incident implementations. As such, this cross-sectional study enabled us to compare implementation of telehealth buprenorphine pre and post enactment of the waiver. Multivariable mixed effects logistic regression was used to assess differences in implementing telehealth buprenorphine inductions by organizational characteristics. We included the census division – Pacific, Mountain, West South Central, West North Central, East North Central, East South Central, New England, Mid-Atlantic and South Atlantic – where the SSP operated as a random effect to account for correlation within these divisions. We first built unadjusted, bivariate models between organization characteristics and whether an SSP implemented telehealth buprenorphine inductions to generate odds ratios (OR), accompanying 95% CI and p-values. If a variable, or any category of a categorical variable, had a p-value < 0.20, we included them in a full model. Variables were retained in the final model if the variable, or any category of a variable, had a p-value < 0.20 in the full, adjusted model. We chose a modest value for variable inclusion so that our approach was more inclusive of potential confounding factors. The full model provided adjusted odds ratios (aOR), corresponding 95% CI and p-values of the associations between organizational characteristics and telehealth buprenorphine implementation at SSPs in 2020. Statistical significance was set at 0.05. All analyses were conducted in Stata v16.1 (College Station, Texas).

## 3. Results

Out of the 421 SSPs, 325 (77.2%) responded to the online survey. Of the 325 SSPs responding to the survey, we excluded 30 that began operating in 2020 or 2021. SSPs providing services in 46 states completed the survey. Overall, 177 (60%) SSPs were non-governmental SSPs, and 118 (40%) SSPs were operated by local, county or state government. The median annual budget for SSPs was $80,000 (Table 1).

In 2020, the incidence of implementing buprenorphine inductions via telehealth was 24% (95% Confidence Interval (CI): 19–30%). Non-governmental SSPs had a higher odds of incident telehealth buprenorphine implementations (aOR = 2.92; 95% CI: 1.22–7.00; p = 0.016), compared to governmental SSPs. SSPs in the Northeast had a higher odds of telehealth buprenorphine implementations, compared to SSPs in the West (aOR = 9.68; 95% CI: 1.20–78.17; p = 0.033). Furthermore, the larger the annual SSP budget, the higher the odds of incident telehealth buprenorphine implementations (aOR = 2.00 per quartile (95% CI: 1.33–2.99; p = 0.001). SSPs located in states with higher OOMRs, higher American Indian or Alaska Native OOMRs, higher non-Hispanic Black OOMRs, higher Hispanic OOMRs and higher non-Hispanic White OOMRs did not have a significantly higher likelihood of telehealth buprenorphine implementation (Table 2).

## 4. Discussion

The temporary waiver of the Ryan Haight Act in March 2020 created the opportunity for SSPs to collaborate with clinicians to provide buprenorphine treatment for OUD using telehealth medicine (Castillo et al., 2020). We found that with just over nine months of the waiver in place, a quarter of United States SSPs had incident buprenorphine implementations via telehealth approaches. The extant scientific literature on telemedicine buprenorphine inductions during COVID have not yielded large scale negative clinical ramifications of providing access virtually (Cole et al., 2020; Mehtani et al., 2021; Tofighi et al., 2021; Buchheit et al., 2021). Together, these findings provide ample evidence that the United States federal government should make this waiver permanent once the COVID state of emergency expires (Jones et al., 2021; Nunes et al., 2021; Davis and Samuels, 2021). It will be imperative for state governments to align with these federal COVID-era policies which mitigate barriers to buprenorphine access. Several states have already let public health emergency orders expire and have reverted to pre-COVID restricted access to this lifesaving medication.

SSPs are ideal venues to integrate buprenorphine treatment – their participants tend to be at high risk for opioid overdose and OUD, and their staff provide culturally competent services to participants (CDC. Syringe, 2020). SSPs are typically community-based and tend to have few to no requirements for people who use drugs to access services. Prior research has shown that participants find their SSP to be a trusted place to receive services where they are treated with respect, in juxtaposition to many experiencing discrimination in traditional healthcare settings (Frost et al., 2021). Many people with OUD require culturally appropriate providers to counteract years of stigma and mistreatment. By partnering with telehealth buprenorphine providers, SSPs can effectively link interested SSP participants with real-time access to appropriate medical providers and support participants to receive their prescriptions. Another study showed that 92% of participants accessing telehealth buprenorphine via SSPs were covered by Medicare or Medicaid (Lambdin et al., 2021). These findings suggest that this approach has the potential to improve equitable access to buprenorphine beyond those with private insurance.

Our results also showed that SSP organizational characteristics were associated with implementation of buprenorphine via telehealth. Non-governmental SSPs had higher odds of implementing buprenorphine through telemedicine than SSPs that are part of local, county, or state government. Qualitative research has shown that during the COVID pandemic, non-governmental SSPs were nimble and innovated to meet increased demands (Wenger et al., 2021). It may be that governmental SSPs are less flexible, more risk averse and/or had fewer staff to engage participants around MOUD, due, in part, to people being reassigned to COVID details, as compared to nongovernmental SSPs (Fernandez-Vina et al., 2020; Maani and Galea, 2020). Regardless of the cause, this finding indicates that despite a growing number of governmental SSPs in the United States, funding for non-governmental SSPs which can be more responsive as new health concerns emerge is critical. Similarly, we found that the higher the budget of the SSP, the higher the odds of having implemented buprenorphine via telehealth. The addition of telemedicine to SSPs likely requires a minimum level of infrastructure for adopting new evidence-based interventions, developing protocols, and nurturing collaborations with medical providers. These findings indicate that increasing funding of SSPs may be necessary to create the infrastructure to facilitate more buprenorphine treatment through telehealth modalities. This would reach a highly vulnerable population that has historically faced disproportionately low access to buprenorphine.

We found that the level of need as measured by the previous years’ opioid overdose mortality rate was not associated with a higher likelihood of telehealth buprenorphine implementation. These findings align with previous results from our 2019 SSP survey, which found that SSPs operating in areas with higher levels of need did not have higher levels of naloxone distribution (Lambdin et al., 2020b). It is alarming that the availability of evidence-based interventions to prevent opioid overdose deaths is not higher where the need for such services is higher. Understanding which state and local initiatives support SSPs to deliver services that meet the underlying need and address disparities is critical.

In addition, we did not observe significant associations between American Indian/Alaska Native or non-Hispanic Black opioid overdose mortality rates and telehealth buprenorphine implementation at SSPs. This finding is particularly concerning as recent studies have shown that non-Hispanic Black and American Indian/Alaska Native individuals have the highest levels and increases in drug overdose mortality rates throughout the United States, (Larochelle et al., 2021; Friedman and Hansen, 2022) and disproportionately low access to buprenorphine treatment (Lagisetty et al., 2019). SSPs tend to be a low barrier setting that can address health disparities among people who have experienced discrimination within other health care settings (Figgatt et al., 2021). Yet, careful analysis should ensure socio-structural dynamics are understood and accommodated to meet the needs for a diversity of communities within SSPs (Lopez et al., 2022).

The study findings should be interpreted with several potential methodological limitations in mind. We do not know the full universe of SSPs in the United States, which precludes us from generalizing to all SSPs. The North American Syringe Exchange Network has been the main resource for SSPs in the United States since 1992. It has held many annual conferences, operates a buyer’s club for SSPs to purchase harm reduction supplies, provides supplies at no charge to emerging and resource limited SSPs, and keeps a list of contact information for all known SSPs in the United States. We used this information to contact SSPs for our survey, yet the possibility exists that there are other SSPs unknown to us. The potential for response bias also exists, since 23% of SSPs did not complete the survey. In particular, it is possible that smaller or underfunded programs were less likely to respond (Centers for Disease Control and Prevention, 2010). However, it is also likely that a portion of non-responders suspended operations or shut down altogether as a result of COVID-19. We did not capture characteristics of the client base of the SSPs to understand how access may have been for clients of different demographic backgrounds including race/ethnicity. All SSP data were self-reported and not validated with official records, making it subject to the possibility of errors. Finally, overdose mortality data are also subject to potential errors in how they were reported.

## 5. Conclusion

This study shows that a substantial number of SSPs implemented buprenorphine telemedicine treatment after the United States federal waiver of the Ryan Haight Act in March 2020. With an increasing number of people with an OUD and rising opioid overdose mortality, (Centers for Disease Control, 2021; Anon, 2015) increased access to evidence-based treatment for OUD is paramount. Given the demand for integrating buprenorphine at SSPs that we observed, exploring other policy and regulatory reform to facilitate integration of other types of MOUD, such as methadone, is warranted. Historically, access to buprenorphine has not only been limited but also less attainable by Black individuals. Increasing availability to the people least likely to have access through primary care practices is best done through SSPs which have minimal barriers for PWID to routinely access them. Our findings suggest that SSPs will increase availability of buprenorphine treatment of OUD if the temporary waiver of the Ryan Haight Act is made permanent. It would be prudent to increase SSP budgets, especially among nongovernmental programs to ensure implementation of this innovative lifesaving treatment.

## Figures and Tables

**Table 1:** Characteristics of syringe service programs operating in the United States as of 2019 (N = 295).

Characteristics	Total

**Program Type**	
Governmental SSP	118 (40)
Non-governmental SSP	177 (60)
**Census Region, n (%)**	
West	132 (45)
Midwest	47 (16)
South	64 (22)
Northeast	52 (18)
**Annual Budget (dollars)**, median (IQR)	80,000 (11,829–230,000)
**Opioid Overdose Mortality Rate**, median (IQR)	17 (8–9)
**American Indian or Alaskan Native Opioid Overdose Mortality Rate,** median (IQR)	8 (7–18)
**Non-Hispanic Black Opioid Overdose Mortality Rate**, median (IQR)	17 (11–20)
**Hispanic Opioid Overdose Mortality Rate**, median (IQR)	10 (5–21)
**Non-Hispanic White Opioid Overdose Mortality Rate**, median (IQR)	20 (9–21)

**Table 2:** Associations of SSP characteristics with telehealth buprenorphine implementation.

Characteristics	OR (95% CI)	p-value	aOR (95% CI)	p-value

**Program Type**				
Governmental SSP	(ref)		(ref)	
Non-governmental SSP	4.87 (2.40–9.90)	< 0.001	2.92 (1.22–7.00)	0.016
**Census Regions**				
West	(ref)		(ref)	
Midwest	1.30 (0.50–3.39)	0.597	3.10 (0.35–21.16)	0.307
South	2.52 (1.16–5.45)	0.019	3.08 (0.44–21.43)	0.255
Northeast	8.04 (3.61–17.92)	<0.001	9.68 (1.20–78.17)	0.033
**Annual Budget (per quartile)**	1.89 (1.29–2.78)	0.001	2.00 (1.33–2.99)	0.001
**Opioid Overdose Mortality Rate** ^a^	0.99 (0.93–1.05)	0.682		
**American Indian or Alaska Native Opioid Overdose Mortality Rate** ^b^	0.98 (0.95–1.02)	0.345		
**Non-Hispanic Black Opioid Overdose Mortality Rate** ^b^	0.99 (0.95–1.02)	0.401		
**Hispanic Opioid Overdose Mortality Rate** ^b^	1.04 (0.96–1.12)	0.367		
**Non-Hispanic White Opioid Overdose Mortality Rate** ^b^	0.97 (0.91–1.02)	0.268		

a 2019.

b 2015–2019.
